# Monte-Carlo-Simulation biometrischer Effektgrößen und deren Einfluss auf das Übersetzungsverhältnis des Hornhautastigmatismus in den Zylinder torischer Intraokularlinsen

**DOI:** 10.1007/s00347-020-01199-y

**Published:** 2020-08-07

**Authors:** Achim Langenbucher, Jens Schrecker, Michael Schwemm, Timo Eppig, S. Schröder, Nóra Szentmáry

**Affiliations:** 1 Institut für Experimentelle Ophthalmologie, Universität des Saarlandes, Kirrberger Str. 100, Gebäude 22, 66424 Homburg, Deutschland; 2 Klinik für Augenheilkunde, Rudolf-Virchow-Klinikum, Glauchau, Deutschland; 3 Dr. Rolf M. Schwiete Zentrum für Limbusstammzellforschung und kongenitale Aniridie, Universität des Saarlandes, Kirrberger Str., Gebäude 22, 66421 Homburg, Deutschland; 4 Klinik für Augenheilkunde, Semmelweis-Universität, Mária u. 39, 1085 Budapest, Ungarn

**Keywords:** Torische Intraokularlinse, Monte-Carlo-Simulation, Linsenberechnung, Vergenzrechnung, Hornhautbrechwert, Vorhersagemodell, Toric intraocular lens, Monte Carlo simulation, Lens power calculation, Vergence propagation, Corneal power, Prediction model

## Abstract

**Hintergrund und Zielsetzung:**

Torische Kapselsacklinsen bieten heutzutage eine zuverlässige Option der permanenten Korrektur eines Hornhautastigmatismus. Zur Ermittlung der für den gewünschten Ausgleich erforderlichen Linsenstärke kann der Operateur entweder auf die in seinem Biometriegerät implementierten Berechnungsmodi oder auf den vom Linsenhersteller angebotenen Kalkulationsservice zurückgreifen. In vielen Fällen wird dabei allerdings keine klassische Linsenberechnung aus biometrischen Daten durchgeführt, sondern nur mit einer vereinfachten Abschätzung gearbeitet, die den Hornhautastigmatismus in den Torus der tIOL übersetzt. Dieses dann zumeist als durchschnittlicher Standardwert genutzte Übersetzungsverhältnis kann jedoch eine erhebliche Schwankungsbreite aufweisen, sodass im ungünstigsten Fall eine Unterkorrektur des refraktiven Zylinders um bis zu 12,5 % oder eine Überkorrektur um bis zu 17 % resultieren kann. Ziel dieser Studie war es aufzuzeigen, welche biometrischen Einflussgrößen das Verhältnis zwischen dem zu korrigierenden Hornhautastigmatismus und dem für dessen Vollkorrektur notwendigen Torus einer Kapselsacklinse bestimmen.

**Methoden:**

Aus der WEB-Plattform IOLCon wurden 16.744 Datensätze extrahiert, und anhand der präoperativen biometrischen Größen und dem postoperativen sphärischen Äquivalent wurde zunächst die axiale Position der Kapselsacklinse formelunabhängig abgeleitet. Anschließend wurde, basierend auf der Propagation sphärozylindrischer Vergenzen, der entsprechende Brechwert einer emmetropisierenden Kapselsacklinse ermittelt. Das Übersetzungsverhältnis als Quotient aus dem Torus der Linse und dem Hornhautastigmatismus wurde mit einer Monte-Carlo-Simulation auf seine potenziellen Einflussgrößen hin untersucht.

**Ergebnisse:**

Die Monte-Carlo-Simulation zeigt, dass nicht von einem konstanten Übersetzungsverhältnis ausgegangen werden kann. Für die hier zugrunde gelegten klinischen Fälle ergibt sich ein mittleres Übersetzungsverhältnis von 1,3938 ± 0,0595 (Median 1,3921) mit einer Spannweite von 1,2131 bis 1,5974. Den größten Einfluss hat hierbei die axiale Position der Kapselsacklinse – je weiter posterior sich diese befindet, desto höher ist das Übersetzungsverhältnis. Aufgrund der Korrelation der axialen Linsenposition mit der Augenlänge kann die Augenlänge als indirekte Einflussgröße gewertet werden. Der Äquivalentbrechwert sowie der Astigmatismus der Hornhaut besitzen keinen nennenswerten Effekt auf das Übersetzungsverhältnis.

**Diskussion:**

In einer ganzen Reihe von Berechnungsmodulen wird die Kalkulation des Torus der Kapselsacklinse dahingehend vereinfacht, dass dieser mittels eines einfachen konstanten Umrechnungsfaktors aus dem gemessenen Hornhautastigmatismus abgeleitet wird. Die vorliegende Studie zeigt jedoch, dass diese Vereinfachung zu deutlich fehlerhaften Ergebnissen führen kann. Dementsprechend wird eine individuelle Berechnung des Torus der IOL aus gemessenen biometrischen Größen (z. B. mittels Vergenzpropagation, Matrizen oder mittels Full-aperture**-**Raytracing) empfohlen.

## Abstract (en)

**Background and objective:**

Toric intraocular lenses (IOL) provide a reliable and predictable option for permanent correction of corneal astigmatism. In order to determine the lens strength necessary for achieving the desired correction, the operator can either use the calculation mode implemented in the biometry device or the calculation service offered by the lens manufacturer; however, in many cases a classical lens calculation from biometric data is not carried out but only a simplified estimation, which translates the corneal astigmatism into the torus of the toric IOL. This translational ratio, which is mostly used as an average standard value, can however show a substantial range of variation, so that in a worst case scenario an undercorrection of the refractive cylinder of up to 12.5 % or an overcorrection of up to 17 % can result. The purpose of this study was to elaborate the biometric effect sizes which determine the relationship between the corneal astigmatism to be corrected and the torus necessary for a full correction of an IOL.

**Methods:**

A total of 16,744 datasets were extracted from the IOLCon web platform and initially the axial position of the IOL implant was derived independent of a formula, based on the preoperative biometric values and the postoperative spherical equivalent. Subsequently, based on a ray propagation strategy for spherocylindrical vergences, the corresponding refractive value of a full correcting toric IOL was calculated. The translational relationship as a ratio between lens toricity and corneal astigmatism was analyzed for potential biometric effect sizes with a Monte Carlo simulation.

**Results:**

The Monte Carlo simulation showed that the ratio of lens toricity to corneal astigmatism cannot be assumed as being constant. The analyzed data revealed an average translational ratio of 1.3938 ± 0.0595 (median 1.3921) with a range from 1.2131 to 1.5974. The axial position of the IOL was found to have the greatest influence, whereby the more posterior the lens position the higher the ratio. Due to the correlation of axial eye length and axial lens position, the eye length can be assumed to be an indirect effect size. The corneal equivalent refractive strength and the corneal astigmatism have no noteworthy effect on the translational ratio.

**Conclusion:**

Many calculation tools on the market simplify toric IOL power calculation by assuming a constant ratio of lens toricity to corneal astigmatism; however, the present simulation study showed that such a simplification can lead to clearly incorrect results. Accordingly, an individual calculation of IOL toricity based on biometric parameters (e.g. based on vergence propagation matrices or full aperture ray tracing) is recommended.

Torische Intraokularlinsen (tIOL) erfreuen sich einer immer größeren Beliebtheit für die Korrektur eines Hornhautastigmatismus. Bei hohem oder exzessivem Hornhautastigmatismus sind tIOL oft die einzige Option der chirurgischen Korrektur. Sie bieten zudem gegenüber Brille oder Kontaktlinse einen deutlich höheren Sehkomfort bei signifikant geringeren optischen Nebenwirkungen (wie z. B. Bildverzerrungen, meridionaler Aniseikonie [12] oder Aberrationen höherer Ordnung bei schrägem Durchblick). Die Indikationsstellung wurde in der vergangenen Dekade auch dahingehend erweitert, dass heute torische IOL bereits zur Korrektur geringer Hornhautastigmatismen ab 0,75 dpt zum Einsatz kommen [3, 5] und damit im Vergleich zu kornealen Inzisionstechniken ein höheres Potenzial an Vorhersagbarkeit und Stabilität ermöglichen. Nicht zuletzt wurde diese Indikationserweiterung durch die Verbreitung von Premiumlinsen mit multifokalen Optiken getriggert, die ihren Nutzen nur dann weitreichend entfalten können, wenn der residuale Astigmatismus gering ist.

Neben torischen Linsen für den Kapselsack werden von einigen Herstellern auch torische Modelle von Zusatzlinsen angeboten, die additiv vor die kristalline Linse (phake tIOL) oder additiv zu einer (möglichst stigmatischen) Kapselsacklinse (Add-on-tIOL) implantiert werden [15]. Letztere werden meist in den Sulcus ciliaris eingesetzt und bieten den Vorteil, dass sie ggf. ohne großen Aufwand nachrotiert, ausgetauscht oder auch wieder entfernt werden können [11, 18]. Nachteilig ist allerdings ihre im Vergleich zu torischen Kapselsacklinsen geringere Rotationsstabilität [8].

Dem Operateur bieten sich verschiedene Optionen für die Berechnung von tIOL. Soll die tIOL so eingesetzt werden, dass die steile Achse der Linse mit dem flachen Meridian der Hornhaut zusammenfällt, so kann eine separate Berechnung eines Linsenbrechwertes für den flachen und den steilen Meridian der Hornhaut erfolgen. Mit diesem Verfahren werden 2 Linsenbrechwerte ermittelt, aus deren Mittelwert und Differenz der Äquivalentbrechwert sowie der Torus der tIOL resultiert. Zu beachten ist hierbei, dass das zum Einsatz kommende Berechnungsverfahren die axiale Position der Linse nicht aus dem Hornhautbrechwert ermittelt, da sonst für beide Hauptschnitte der Hornhaut unterschiedliche axiale Positionen der tIOL resultieren, was wenig sinnhaft wäre. Soll die tIOL jedoch mit ihrer steilen Achse abweichend vom flachen Hornhautmeridian implantiert werden (um z. B. gezielt die vorbestehende Achse in der Refraktion zu drehen) oder soll der Effekt einer nicht perfekt ausgerichteten tIOL auf die zu erwartende Refraktion nach dem Eingriff abgeschätzt werden, so liegen gekreuzte Zylinder vor, und das zuvor beschriebene vereinfachte Vorgehen ist nicht mehr anwendbar. Zum Einsatz kommen dann Berechnungsverfahren, die mit sphärozylindrischen Vergenzen umgehen können, oder erweiterte Verfahren auf der Basis von 4 × 4-Translations- und Refraktionsmatrizen [12].

In der Regel wird praktisch von allen Herstellern torischer Kapselsacklinsen ein Berechnungsservice angeboten. Hierzu werden die biometrischen Daten des Patienten anonymisiert an den Linsenhersteller übermittelt, und aus den Daten (wie Krümmungsradius bzw. Brechwert der Hornhaut im flachen und steilen Meridian, Augenlänge, ggf. phake Vorderkammertiefe, Linsendicke und horizontaler Hornhautdurchmesser) wird die torische Kapselsacklinse ermittelt. In vielen Fällen wird dabei aber keine klassische Linsenberechnung aus den biometrischen Daten durchgeführt, sondern ausschließlich eine Berechnung des Linsentorus. Das bedeutet, dass der Operateur das in seinem Biometer implementierte Berechnungsschema nutzt, um die stigmatische Äquivalentlinse zu berechnen. Über den Berechnungsservice des Herstellers erhält er nach Übermittlung der Äquivalentlinse und der Brechwerte der beiden Hornhautmeridiane eine Empfehlung für den Linsentorus. Bei einigen Herstellern wird dabei mit einer vereinfachten Abschätzung gearbeitet, die den Hornhautastigmatismus in den Torus der tIOL überführt.

Ziel der vorliegenden Arbeit ist es, mit einer Monte-Carlo-Simulation das Übersetzungsverhältnis (Quotient) von Torus der tIOL und Astigmatismus der Hornhaut auf seine Einflussgrößen hin zu untersuchen.

## Methoden

Als Datenbasis für die Erstellung des Monte-Carlo-Simulationsmodells diente die IOLCon-Plattform (iolcon.org), auf der von uns eine Übersicht technischer Daten, Lieferbereiche sowie herstellerempfohlene und von uns optimierte Formelkonstanten für die Anwendung in der Ophthalmologie zur Verfügung gestellt werden [19]. Die von klinischen Zentren oder von Linsenherstellern im Rahmen von Studien erhobenen klinischen Daten wurden entsprechend den Nutzungsvereinbarungen herangezogen, um eine Verteilungscharakteristik der biometrischen Größen zu entwickeln sowie mögliche Zusammenhänge zu untersuchen. Insgesamt wurden 16.744 konsekutive klinische Daten für die Monte-Carlo-Simulation [7] verwendet, bei denen der Krümmungsradius der Hornhaut in beiden Hauptmeridianen, die optisch gemessene Augenlänge, die phake Vorderkammertiefe (gemessen vom Apex der Hornhaut bis zum vorderen Scheitel der kristallinen Linse) sowie die postoperative Refraktion mit Sphäre und Zylinder auswertbar waren. Auf der Basis einer Vergenzbetrachtung für sphärozylindrische Vergenzen wurde unabhängig von etablierten Linsenberechnungsstrategien (Formelberechnung nach z. B. Haigis, Holladay 1, Hoffer Q, SRK/T [6]) die Position einer „dünnen“ Kapselsacklinse aus den biometrischen Daten und der gemessenen postoperativen Refraktion abgeleitet. Dazu wurde von folgenden Annahmen bzw. Abschätzungen ausgegangen:

- Die Vergenz in der Ebene der Brillenkorrektur beträgt −1/6 dpt (Das heißt, die Erhebung der postoperativen klinischen Daten erfolgte mit einer Refraktionsmessstrecke von 6 m).
- Der Hornhautscheitelabstand wurde mit 12 mm angenommen.
- Der Brechwert der Hornhaut in den beiden Hauptschnitten wurde mit einem Keratometerindex von 1,332 aus den im Datenpool hinterlegten Hornhautkrümmungsradien ermittelt.
- Die tIOL wurde als dünne Linse angenommen. (Dies ist gleichbedeutend mit der Annahme, dass der Äquivalentbrechwert sowie der Torus der tIOL in Dioptrien angegeben werden und geometrische Daten sowie der Brechungsindex der tIOL nicht zur Verfügung stehen.)
- Die Brennebene des Auges liegt bei der Ermittlung der postoperativen Refraktion in einem Abstand hinter dem Hornhautapex, der der Augenlänge entspricht. (Das heißt, hinter der als dünne Linse angenommenen Hornhaut ist die tIOL im Abstand der effektiven Linsenposition [ELP] positioniert. Nach der ebenfalls als dünne Linse angenommenen tIOL [AL-ELP] folgt Glaskörperstrecke bis zur Brennebene.)

Für jeden Einzelfall wurde aus den präoperativ erhobenen biometrischen Daten und der aus der postoperativen Refraktion (sphärisches Äquivalent) rückgerechneten ELP eine tIOL berechnet, die den Hornhautastigmatismus vollständig ausgleicht. Der Torus der tIOL wurde dann zum Astigmatismus der Hornhaut ins Verhältnis gesetzt und als „Übersetzungsverhältnis“ definiert. Anschließend erfolgte eine Analyse des Einflusses biometrischer Größen (Augenlänge, Äquivalentbrechwert der Hornhaut, Hornhautastigmatismus, geschätzte Linsenposition ELP) auf dieses Übersetzungsverhältnis. Des Weiteren wurde der Zusammenhang zwischen der ELP und der Augenlänge sowie der Zusammenhang des Äquivalentbrechwertes der tIOL mit der Augenlänge und der geschätzten Linsenposition untersucht.

Für die Berechnungen, die statistische Auswertung der Regression sowie die grafische Aufarbeitung wurde die Interpretersprache Matlab (The MathWorks, Version 2019b) verwendet. Bei der Beschreibung der Zusammenhänge kam ein linearer Regressionsansatz zum Einsatz. Neben den Koeffizienten der linearen Regression wurden jeweils der korrigierte quadrierte Wert für R, das Signifikanzniveau *p* sowie der Standardfehler SE angegeben. Gemäß der Definition von Cohen [4] wurde ein Zusammenhang als klein oder gering betrachtet, wenn 0,1 < |R| ≤ 0,3 gilt, ein moderater Zusammenhang ist durch 0,3 < |R| ≤ 0,5 gegeben und ein großer Zusammenhang durch |R| > 0,5.

## Ergebnisse

Das Übersetzungsverhältnis (Zylinder der tIOL: Hornhautastigmatismus) zeigt innerhalb der zugrunde gelegten klinischen Fälle eine Spannweite zwischen 1,2131 und 1,5974 (Mittelwert: 1,3938 ± 0,0595; Median: 1,3921). Es kann daher prinzipiell nicht von einem konstanten Übersetzungsverhältnis ausgegangen werden.

**Figure 1:**
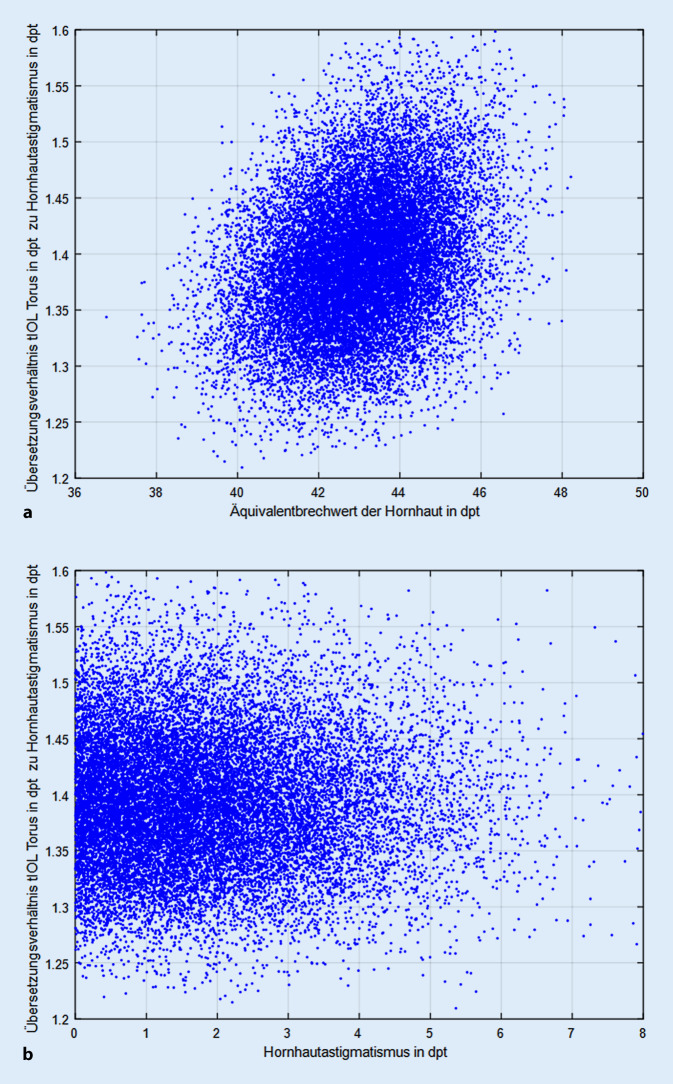

Die Abb. 1a zeigt die Abhängigkeit des Übersetzungsverhältnisses vom Äquivalentbrechwert der Hornhaut und Abb. 1b die Abhängigkeit vom Hornhautastigmatismus. Es kann direkt abgelesen werden, dass das Übersetzungsverhältnis nur gering vom Äquivalentbrechwert der Hornhaut und nicht vom Hornhautastigmatismus systematisch beeinflusst wird. Die Regressionsgerade aus Abb. 1a ist definiert durch y = 0,8786 + 0,0116 × mit einem Standardfehler von 0,0116 für den Achsenabschnitt und 0,0003 für x, das korrigierte R^2^ beträgt 0,0897 (R = 0,2994). Die Regressionsgerade aus Abb. 1b ist definiert durch y = 1,3932 + 0,0000 × mit einem Standardfehler von 0,0007 für den Achsenabschnitt und 0,0003 für x, das korrigierte R^2^ ist kleiner als 0,0001 (R < 0,1).

**Figure 2:**
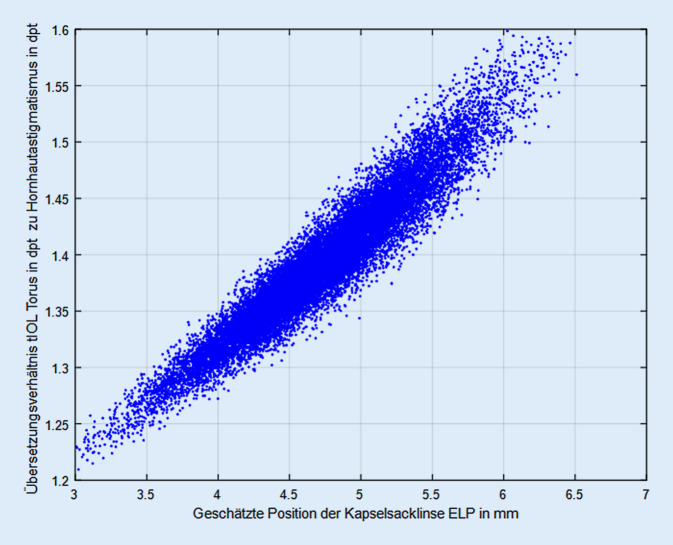

Die Abb. 2 stellt die Abhängigkeit des Übersetzungsverhältnis von der geschätzten Position der Kapselsacklinse relativ zum Hornhautscheitel dar. Hier besteht ein starker Zusammenhang, der aufzeigt, dass bei größeren Werten für die Linsenposition (weiter posterior gelegene tIOL) das Übersetzungsverhältnis deutlich ansteigt. Die Regressionsgerade aus Abb. 2 ist definiert durch y = 0,8917 + 0,1061 × mit einem Standardfehler von 0,0011 für den Achsenabschnitt und 0,0002 für x, das korrigierte R^2^ liegt bei 0,91 (R = 0,9539).

**Figure 3:**
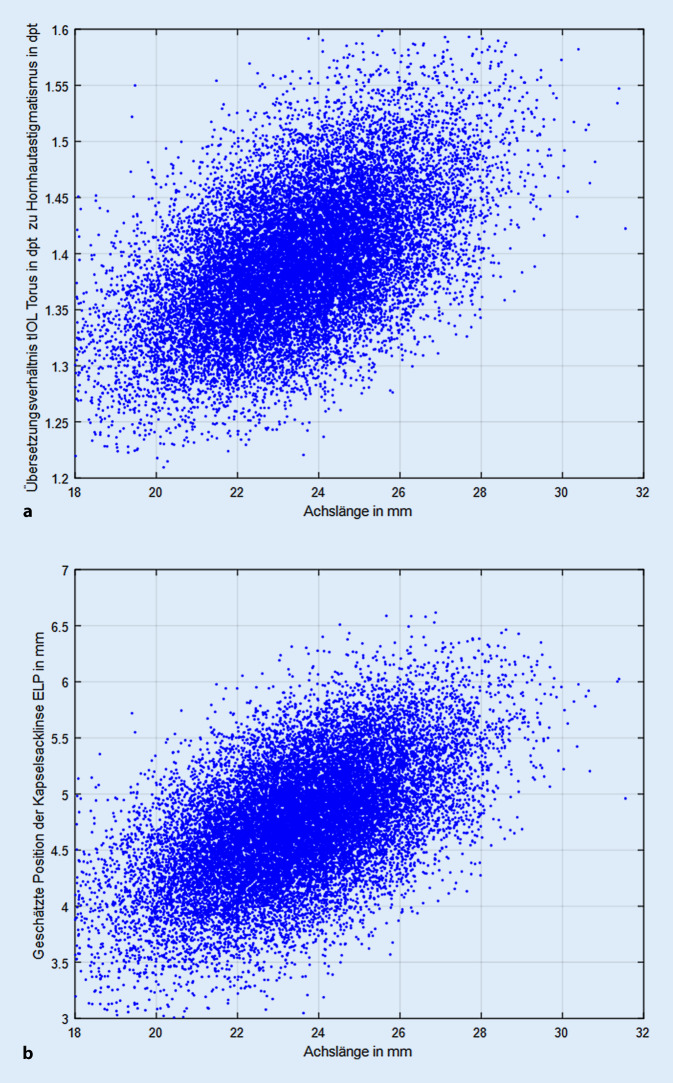

Die Abb. 3a dokumentiert die Abhängigkeit des Übersetzungsverhältnis von der Augenlänge. Hier besteht ein starker Zusammenhang, der aufzeigt, dass bei größeren Werten für die Augenlänge (tendenziell myope Augen) auch das Übersetzungsverhältnis ansteigt. Die Regressionsgerade aus Abb. 3a ist definiert durch y = 1,0198 + 0,0159 × mit einem Standardfehler von 0,0043 für den Achsenabschnitt und 0,0002 für x, das korrigierte R^2^ liegt bei 0,276 (R = 0,5254). Die Abb. 3b macht deutlich, dass der unter 3a beschriebene Zusammenhang zwischen dem Übersetzungsverhältnis und der Augenlänge der Korrelation zwischen Augenlänge und geschätzter Linsenposition angelastet werden kann. So wird sich im Allgemeinen bei Augen mit einer großen Augenlänge die tIOL eher in einer größeren Entfernung hinter dem Hornhautscheitel positionieren. Die Regressionsgerade aus Abb. 3b ist definiert durch y = 1,1772 + 0,1513 × mit einem Standardfehler von 0,0379 für den Achsenabschnitt und 0,0016 für x, das korrigierte R^2^ liegt bei 0,306 (R = 0,5532).

**Figure 4:**
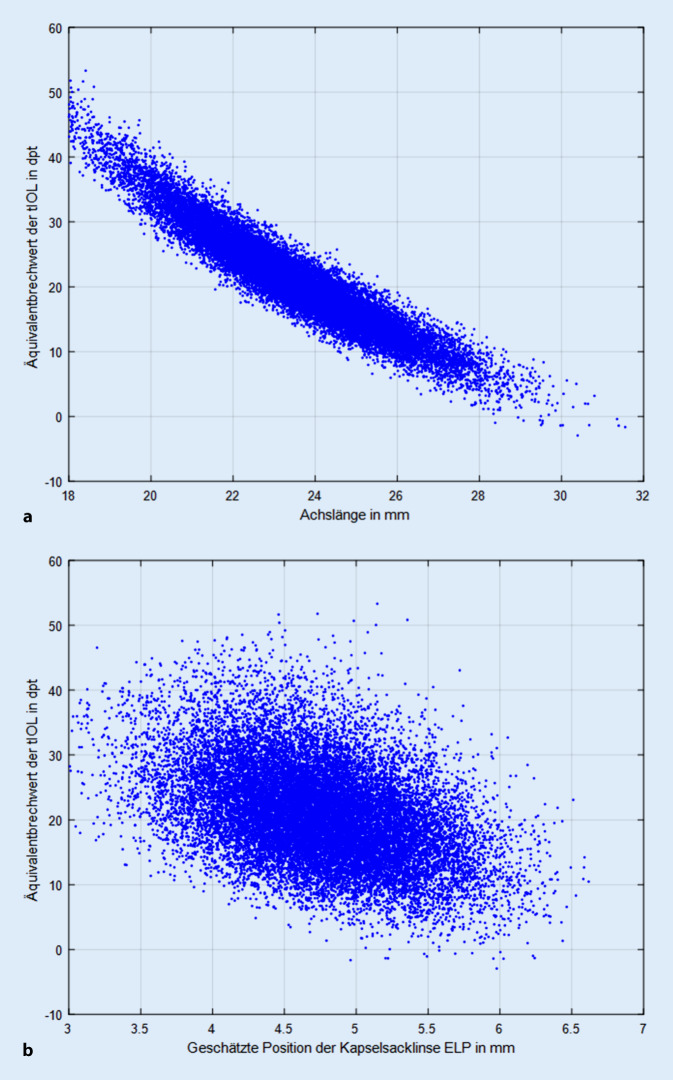

Die Abb. 4a–c zeigt den Zusammenhang zwischen dem Äquivalentbrechwert der vollkorrigierenden tIOL zur Augenlänge (Abb. 4a), zur geschätzten Position der Kapselsacklinse relativ zum Hornhautscheitel (Abb. 4b) sowie zum Äquivalentbrechwert der Hornhaut (Abb. 4c). Aus den Definitionen der entsprechenden Regressionsgeraden kann man sehr einfach die Empfindlichkeit des Äquivalentbrechwertes der tIOL (der „Linsenstärke“) in Bezug auf biometrische Größen (bzw. deren Fehlbestimmung) eruieren. Die Regressionsgerade aus Abb. 4a ist definiert durch y = 105,5 − 3,5942 × mit einem Standardfehler von 0,2044 für den Achsenabschnitt und 0,0087 für x, das korrigierte R^2^ liegt bei 0,896 (R = 0,9466). Dies bedeutet, dass für jeden Millimeter, den die Augenlänge zunimmt, der Brechwert der emmetropisierenden tIOL um rund 3,6 dpt zu reduzieren ist. Die Regressionsgerade aus Abb. 4b ist definiert durch y = 49,151 − 5,9283 × mit einem Standardfehler von 0,4281 für den Achsenabschnitt und 0,00897 für x, das korrigierte R^2^ liegt bei 0,179 (R = 0,4231). Dies zeigt, dass pro Millimeter, den die Position der Kapselsacklinse weiter posterior positioniert ist, der Brechwert der emmetropisierenden tIOL um rund 5,9 dpt zu reduzieren ist. Dieser Zusammenhang zwischen der geschätzten axialen Position der Kapselsacklinse und dem Äquivalentbrechwert der tIOL stellt eine Überlagerung verschiedener Effekte dar und resultiert v. a. aus dem Zusammenhang zwischen der Augenlänge und der geschätzten axialen Linsenposition. Dekorrelliert man den Effekt der Abhängigkeit des Äquivalentbrechwertes der tIOL mit einer multiplen linearen Regression mit den Effektgrößen der geschätzten axialen Position der Kapselsacklinse relativ zum Hornhautapex und der Augenlänge, ergibt sich eine Regressionsgerade der Form y = 103,1 + 1,9163 x1 − 3,8766 x2, wobei x1 die axiale Linsenposition und x2 die Augenlänge bezeichnet. Die Standardfehler für den Achsenabschnitt / x1 / x2 liegen bei 0,1943 / 0,0358 / 0,0097, das korrigierte R^2^ bei 0,91 (R = 0,9539). Das bedeutet, dass für jeden Millimeter, den die Kapselsacklinse weiter posterior geschätzt wird, der Äquivalentbrechwert der tIOL um 1,9 dpt ansteigt und für jeden Millimeter, den die Augenlänge zunimmt, der Äquivalentbrechwert der tIOL um etwa 3,9 dpt abnimmt. Die Regressionsgerade aus Abb. 4c ist definiert durch y = 81,32 − 1,4009 x mit einem Standardfehler von 1,4644 für den Achsenabschnitt und 0,0341 für x, das korrigierte R^2^ liegt bei 0,0781 (R = 0,2795). Dies veranschaulicht, dass für jede Dioptrie, die der Äquivalentbrechwert der Hornhaut zunimmt, der Brechwert der emmetropisierenden tIOL um rund 1,4 dpt zu reduzieren ist.

**Figure 5:**
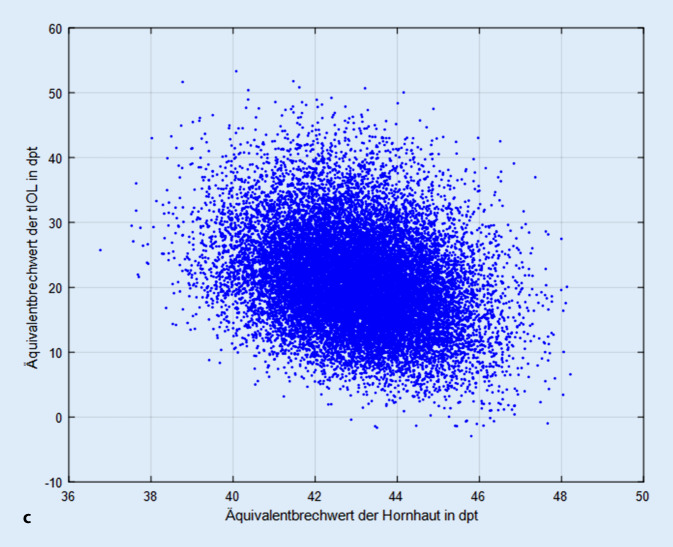

## Diskussion

Seit der ersten Implantation einer torischen Kapselsacklinse im Jahr 1992 [20] steigt der Anteil torischer Linsen bei der Kataraktchirurgie stetig an. Jedoch erfolgt auch heute nicht in jedem Fall, bei dem eine torische Optik von Vorteil für den Patienten wäre, die Implantation einer entsprechenden Intraokularlinse [10]. Speziell bei Premiumlinsen mit multifokaler Optik ist es wichtig, dass bereits ein Hornhautastigmatismus von 1 dpt (besser ab 0,75 dpt) mit der IOL korrigiert wird, um dem Patienten den maximalen Nutzen der Multifokalität zu ermöglichen [2, 21, 22]. Die Brechkraft der für eine Vollkorrektur notwendigen torischen IOL kann mithilfe eines Umrechnungsfaktors von dem auszugleichenden Hornhautastigmatismus abgeleitet werden.

Der Faktor, also das „Übersetzungsverhältnis“, ist jedoch nicht konstant und wurde nach unserem Kenntnisstand in der Literatur bisher nicht konsequent untersucht [13]. Für den Ophthalmochirurgen wäre es jedoch von Vorteil, wenn bei der präoperativen Planung die Bandbreite, die dieses Übersetzungsverhältnis annehmen kann, und deren Einflussgrößen bekannt sind. Nach unserer Auswertung einer sehr großen Anzahl biometrischer Daten von Kataraktpatienten liegt das Übersetzungsverhältnis im Mittel bei ca. 1,4 – die Bandbreite reicht jedoch von 1,2 bis 1,6. Im ungünstigsten Fall kann so bei vereinfachter Annahme eines Standardwertes von 1,4 der refraktive Zylinder um 12,5 % unterkorrigiert oder um 17 % überkorrigiert werden. Den größten Einfluss auf das Übersetzungsverhältnis hat dabei die Position der Kunstlinse: Je weiter posterior sich die tIOL im Auge stabilisiert, desto größer ist das Übersetzungsverhältnis bzw. bei einem vorgegebenen Torus desto geringer ist die Korrektur des Hornhautastigmatismus. Für die Augenlänge besteht ein indirekter Zusammenhang, da die geschätzte axiale Linsenposition positiv mit der Augenlänge korreliert. Bei großen Bulbuslängen (meist myope Augen) ist in der Regel auch der vordere Augenabschnitt entsprechend größer, und die Kapselsacklinse wird sich weiter posterior im Auge stabilisieren. Der mittlere Brechwert der Hornhaut hat nur einen geringen und der Hornhautastigmatismus keinen Einfluss auf das Übersetzungsverhältnis.

Soll das Übersetzungsverhältnis durch eine einfache lineare Regression beschrieben werden, so sollte an erster Stelle die geschätzte axiale Linsenposition (ELP) herangezogen werden, die den größten Teil der Varianz erklärt. Wird der Regressionsansatz noch um die Augenlänge erweitert (im Sinne einer multiplen Regression), sollte eine Beschreibung des Übersetzungsverhältnisses mit für klinische Anwendungen ausreichender Genauigkeit möglich sein.

In einer Reihe von Produktinformationen zu torischen Linsen finden sich Empfehlungen derart, dass sich ein torisches IOL-Modell mit z. B. einem Torus von 4,5 dpt für die Korrektur eines Hornhautastigmatismus von 3,0–3,75 dpt eignet. Ebenso wird in einigen Online-Kalkulatoren für torische Linsen ein fester Wert für das Übersetzungsverhältnis bzw. ein vereinfachtes Nomogramm bei der Auswahl der „geeigneten“ torischen Linse zugrunde gelegt [9, 16, 17]. Aus den Auswertungen der vorliegenden Arbeit wird aber deutlich, dass eine derartige Zuordnung nur ungenau möglich ist, da mehrere biometrische Größen – v. a. die axiale Position der tIOL – sich entscheidend auf das Übersetzungsverhältnis auswirken. Dementsprechend sollte auch bei der Auswahl bzw. dem Einsatz von Kalkulationsschemata für torische Kapselsacklinsen sehr sorgfältig darauf geachtet werden, dass „intern“ nicht vereinfachend ein festes Übersetzungsverhältnis hinterlegt ist, sondern (basierend auf den gemessenen biometrischen Größen des Auges) der Torus der zu implantierenden tIOL individuell ermittelt wird.

Die hier für Kapselsacklinsen dargestellten Ergebnisse lassen sich nicht unmittelbar auf das Übersetzungsverhältnis torischer Zusatzlinsen übertragen. So fällt im Mittel das Übersetzungsverhältnis für Zusatzlinsen (die vor die kristalline Linse oder vor eine stigmatische Kapselsacklinse in den Sulkus oder in die Vorderkammer gesetzt bzw. irisgestützt fixiert werden) in der Regel geringer aus, da hier die Korrektur näher an der Hornhautebene erfolgt.

Arbeitet man mit einer Berechnungsstrategie auf der Basis von sphärozylindrischen Vergenzen oder Matrizen oder mit Full-aperture-Raytracing, so ergibt sich (bei einer zuverlässigen empirischen Abschätzung der axialen Linsenposition) das Übersetzungsverhältnis per se, da die sphärozylindrische Vergenz, ausgehend von der Brillenebene, sukzessive zur jeweils nachgeschalteten Ebene mit einer refraktiven Grenzfläche durch ein homogenes optisches Medium verfolgt wird und sich damit die sphärozylindrische Korrektur, die eine tIOL zu leisten hat, in jeder beliebigen Position angeben lässt.

Die vorliegenden Auswertungen der Monte-Carlo-Simulation erlauben für eine Abschätzung des Übersetzungsverhältnisses aus Torus der Kapselsacklinse zum Hornhautastigmatismus folgende Option:

Übersetzungsverhältnis ≈0,8917 + 0,1061 geschätzte axiale Position der Kapselsacklinse in mm.

Weiterhin kann aus der Simulation eine Faustformel für eine vereinfachte Abschätzung der Empfindlichkeit des Äquivalentbrechwertes der tIOL gegenüber den Messgrößen Augenlänge und der geschätzten axialen Position der Kapselsacklinse abgeleitet werden:

Für jeden Millimeter Zunahme der Augenlänge reduziert sich der Äquivalentbrechwert der tIOL um 3,9 dpt, für eine posteriore Verlagerung der Kapselsacklinse um 1 mm erhöht sich der Äquivalentbrechwert der emmetropisierenden Linse um 1,9 dpt.

Allerdings unterliegt die hier vorgestellte Monte-Carlo-Simulation einigen Einschränkungen: So wurden aus dem Datenpool der IOLCon-Plattform, die als breite Datenbasis neben technischen Spezifikationen und Lieferbereichen von über 400 Intraokularlinsenmodellen (von 25 Linsenherstellern) den Ophthalmochirurgen die vom Hersteller empfohlenen Formelkonstanten sowie die Ergebnisse der Konstantenoptimierung zur Verfügung stellt, Daten von über 16.000 Patienten mit operationswürdiger Katarakt für die Auswertung herangezogen. Die jeweiligen Messbedingungen für die Erhebung der postoperativen Refraktion sind dabei allerdings nicht standardisiert. So liegen uns zwar in vielen Fällen Daten über die Messdistanz vor, in anderen Fällen mussten wir jedoch von der Annahme ausgehen, dass die Messbedingungen konform zum ISO-Standard eingehalten wurden. Zwischen den gemäß ISO-Standard für die Refraktionsbestimmung zulässigen Grenzen von 4–6 m liegt ein Offsetfehler von 1/12 dpt, der direkt die Abschätzung der axialen Linsenposition verfälschen kann. Wir gingen bei unserer Simulation primär von einer Messdistanz von 6 m aus. In den Fällen, bei denen uns eine abweichende Messdistanz bekannt war, wurde die Refraktion an die Messdistanz von 6 m angepasst. Die Datenbasis der in der IOLCon-Plattform hinterlegten klinischen Daten ist nicht primär auf torische Kapselsacklinsen ausgelegt, sondern umfasst klinische Daten sowohl für stigmatische als auch für torische oder Multifokallinsen. Möglicherweise weichen die biometrischen Charakteristika einer Population, die speziell zur Implantation einer torischen Kapselsacklinse ansteht, von den hier zugrunde gelegten in der Form ab, dass die Verteilung der Werte für den Hornhautastigmatismus hin zu höheren Amplituden verschoben ist. Für die Betrachtung könnten die Streudiagramme geringfügig verzerrt werden, die Kernaussage dieser Arbeit bleibt jedoch vollumfänglich bestehen.

Zusammenfassend lässt sich sagen, dass für die vollständige Korrektur eines Hornhautastigmatismus mit einer torischen Kapselsacklinse der Hornhautastigmatismus mit einem variablen Faktor zwischen 1,2 und 1,6 (im Mittel 1,4) multipliziert werden muss. Dieses Übersetzungsverhältnis hängt wesentlich von der axialen Position der torischen Linse im Auge ab, jedoch nur unwesentlich vom Äquivalentbrechwert der Hornhaut oder dem Hornhautastigmatismus. Im Rahmen der präoperativen Kalkulation torischer IOL sollte unbedingt beachtet werden, ob das verwendete Berechnungsschema nur einen festen Faktor oder einen an die individuellen biometrischen Größen angepassten Algorithmus verwendet.
